# Alzheimer’s Disease Research Using Human Microglia

**DOI:** 10.3390/cells8080838

**Published:** 2019-08-05

**Authors:** Lih-Fen Lue, Thomas G. Beach, Douglas G. Walker

**Affiliations:** 1 Banner Sun Health Research Institute, Sun City, AZ 85351, USA; 2 Neurodegenerative Disease Research Center and School of Life Sciences, Arizona State University, Tempe, AZ 84027, USA; 3 Molecular Neuroscience Research Center, Shiga University of Medical Science, Otsu 520, Japan

**Keywords:** neuroinflammation, microglia, cell culture, brain, amyloid, neurodegeneration, autopsy

## Abstract

Experimental studies of neuroinflammation in Alzheimer’s disease (AD) have mostly investigated microglia, the brain-resident macrophages. This review focused on human microglia obtained at rapid autopsies. Studies employing methods to isolate and culture human brain microglia in high purity for experimental studies were discussed. These methods were employed to isolate human microglia for investigation of a number of features of neuroinflammation, including activation phenotypes, neurotoxicity, responses to abnormal aggregated proteins such as amyloid beta, phagocytosis, and the effects of aging and disease on microglia cellular properties. In recent years, interest in human microglia and neuroinflammation has been renewed due to the identification of inflammation-related AD genetic risk factors, in particular the triggering receptor expressed on myeloid cells (TREM)-2. Because of the difficulties in developing effective treatments for AD, there has been a general need for greater understanding of the functions of microglia in normal and AD brains. While most experimental studies on neuroinflammation have employed rodent microglia, this review considered the role of human microglia in experimental studies. This review focused on the development of in vitro methodology for the culture of postmortem human microglia and the key findings obtained from experimental studies with these cells.

## 1. Introduction 

Alzheimer’s disease (AD) is the major cause of cognitive decline and dementia in the elderly. To date, new treatments, particularly those aimed at removing or preventing amyloid-beta (Aβ) accumulation, have been clinically ineffective in preventing the loss of cognition [1,2,3]. Because of the need to identify effective treatments for AD, neuroinflammation has become a renewed research interest.

One of the most striking late 20th-century findings in neuropathological studies of AD was the large increase in numbers of microglia with an activated morphology and an increased expression of the major histocompatibility complex class II (MHC-II) protein HLA-DR in areas of neurodegeneration [4,5,6]. From these early studies, the “inflammatory hypothesis” of AD was developed, suggesting that excessive microglial production of cytotoxic cytokines, reactive oxygen species (ROS), or degradative enzymes could be early events in AD pathogenesis. Even if microglial activation was not a primary event, but rather a reaction to Aβ plaques or other toxic protein, such activation has been thought to be capable of accentuating ongoing neuronal death.

## 2. Development and Limitations of Methodology for Isolating Human Brain Microglia

### 2.1. Sources of Human Brain Tissue

The authors have been studying human microglia isolated from autopsy brains for over 25 years [7,8]. Our focus was on mechanisms of neuroinflammation in AD, particularly the interactions of microglia with Aβ peptides [9]. Our studies established the feasibility of developing experimental models using human microglia derived from elderly and diseased autopsy brains when such material is available. We cannot claim to be the first to do this, as a paper employing human autopsy brains for isolating white matter microglia was published by Hayes et al. from the United Kingdom in 1988 [10], a few years before the authors’ first papers [7,8]. Human microglial cultures derived from alternative sources than autopsy brain tissue have also been used in experiments by other groups. Alternative sources of tissue include surgically-excised or fetal brain tissues [11,12,13,14,15,16,17,18,19,20,21], however, the availability of such brain tissues is more limited than autopsy material and its acquisition for research purposes involves multiple layers of human ethical approval and informed consent.

The availability of human autopsy brain tissue is potentially greater for a larger number of researchers, but this tissue is optimal when available through a rapid autopsy program. The authors are associated with such a program in Sun City, Arizona, the Banner Sun Health Research Institute (BSHRI) Brain and Body Donation Program [22], where consented subjects undergo autopsy with a median postmortem delay of 3.2 h. The Netherlands Brain Bank (NBB) has a similar program [23,24,25,26]. Both programs have normal donors as well as donors with diagnoses of various neurological diseases. Researchers have utilized isolated and cultured microglia from human autopsy brains from these brain banks for experimental studies.

### 2.2. Development of Microglial Isolation and Primary Culture Methods from Human Brains

**Figure 1:**
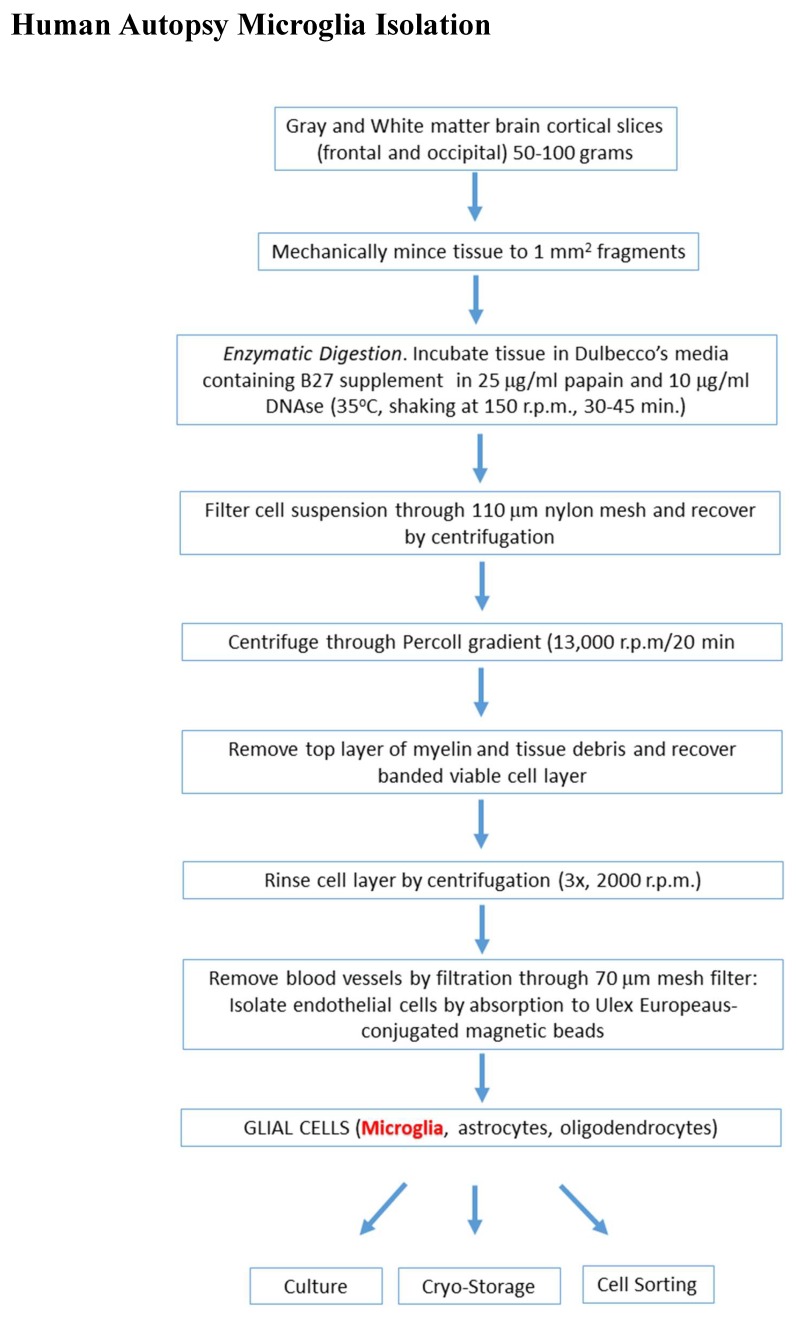
Detailed scheme outlining protocol used to isolate microglia and other non-neuronal cells from rapid autopsy postmortem brain tissue.

Methods for isolating microglia from human brain tissue generally involve enzymatic digestion of a portion of brain tissue with trypsin, collagenase or papain, followed by Percoll (or similar) density gradient centrifugation to separate the myelin and erythrocytes from the cells present in the digested tissue. This approach was initially developed to isolate oligodendrocytes from human brains [27] but was subsequently shown to be useful for isolating microglia [7,8]. The procedure that the authors used with minor changes during last 25 years to obtain microglia (and other cell types) is illustrated in Figure 1. The isolated cell layer from enzymatically processed brain tissue contains microglia, astrocytes, oligodendrocytes, brain endothelial cells, blood vessel fragments of various sizes, and non-viable neurons. From this basic method, pure microglia can be obtained by their characteristic strong and rapid attachment to plastic culture surfaces (1–4 h), while the other cell types require coating matrices to attach. After an attachment period for microglia, non-adherent cells are removed and transferred for further selection of other cell types. Although earlier publications have employed trypsin for tissue digestion, in recent years, we and others have substituted with the enzyme papain [28,29,30]. Since year 2018, we have also added procedures to cryoprotect mixed glia cells and cell selection.

Using papain instead of trypsin, we found that the isolated cell layer after the Percoll gradient centrifugation step in our protocol contains viable brain endothelial cells and blood vessel fragments that can be the source of pericytes and smooth muscle cells, along with a greater yield of microglia and astrocytes. If employing papain, vessel-associated cells must be removed prior to in vitro culture of microglia or the purity of the cultures is reduced. For this purpose, we retrieved brain microvascular endothelial cells using affinity to Ulex Europaeus Agglutinin I (Vector Laboratory, Burlingame, CA, USA) conjugated to magnetic beads using a CELLection Biotin Binder kit (Thermo Fisher Scientific, Waltham, MA, USA), while pericytes and smooth muscle cells were isolated by culturing blood vessel fragments on collagen-coated plates [31]. By adding these additional steps, the purity of microglia in culture can be >99% [32]. The advantages of using these traditional methods are the lower cost for producing the same number of microglia compared to cell-sorting methods. With this basic method, fluorescence-conjugated or magnetic bead-conjugated antibody reagents are not needed, nor the specialized equipment or reagents. Cell-sorting procedures usually require preparing cells at a high density, e.g., 1 million cells/µL, which has consequences on cell viability if culturing is a requirement. Overall, special caution is required to prevent the loss of the viability of the sorted cells. This is not an issue if direct culture is to be carried out. Cell-sorting methods have advantages if the microglia are to be analyzed immediately after isolation, however, with these immunological sorting methods, the amount of tissue that can be processed is limited, as is the number of recovered cells. Fewer than 1 million microglia were reported to be recovered by cell-sorting from one gram of white or grey matter tissue [26]. To generate a sufficient number of human microglia for rigorously designed experimentation involving multiple replicates and treatments with pro- and anti-inflammatory reagents, the traditional method has proven more feasible to obtain sufficient numbers of cells.

Variations on this procedure have also been developed by other researchers. Researchers associated with the NBB have developed separate isolation procedures for cortical gray matter and corpus callosum white matter [26]. For gray matter (occipital cortex), the tissue was first mechanically dissociated, then digested with collagenase followed by gradient separation in Percoll. For the preparation of white matter microglia derived from corpus callosum, they utilized trypsin digestion, Percoll separation and adherence to unmodified plastic surfaces. Some researchers have shown that enzyme digestion followed by plating without Percoll separation does enable the adherence of microglia [30,33]. This method can be efficient with small amounts of gray matter tissue (temporal lobe resection tissue) or fetal brain material with limited amounts of myelin but excessive amounts of myelin interfere with microglia attachment. There is an experimental reason for separating cells from myelin, as myelin can have significant activation properties that affect the resting phenotypes of isolated microglia [14]. An alternative isolation protocol used digestion with trypsin, followed by two-step purification through 35% sucrose, followed by Ficoll/sodium diatrozite gradient centrifugation [34]. The traditional methods described above have more recently been supplemented with the use of fluorescent-activated or magnetic-activated cell sorting (FACS or MACS) to separate different classes of brain cells based on their expression of cell surface-specific markers. Using sorting technology, it is not only possible to isolate human brain microglia at high purity for subsequent in vitro culture, but also to directly analyze microglia ex vivo from the brain [35,36].

### 2.3. Other Tissue Factors (Gray Matter Compared to White Matter)

The successful isolation of microglia from gray compared to white matter has varied depending on the investigators. Microglia isolation employing MACS technology to select microglia cells from gray matter compared to white matter using CD11b antibody-labeled magnetic microbeads has been reported [26,37]. The mean CD11b positive microglia cell yield from occipital gray matter was 2.4-fold greater than from corpus collosum. Despite the greater yield of microglia from gray matter, a difference in the properties of isolated microglia was reported. White matter microglia isolated using this method attached to culture surfaces and could be studied in vitro, while cortical gray matter microglia showed no adherence after 2 days in culture. As AD pathology mainly involves gray matter, our protocol utilized brain tissue containing both gray matter and white matter. There is considerable difficulty in precisely separating gray from white matter along the cortical convolutions, which can lead to wastage of valuable human brain tissue. As routine, we used both total gray and white matter from frontal and occipital cortex, consisting of approximately 40% gray matter and 60% white matter for the experimental isolation of microglia and other cells. Using our reported protocol (Figure 1), we did not observe any difficulty with non-adherence of microglia. Based on our experience, the agonal state of the donor is the most critical factor in isolating viable cells. There are no data from our studies supporting the advantages of separated gray or white matter as starting material for experimental purposes. However as the morphology and activation features of white matter microglia are noticeably different from pathology-associated gray matter microglia in fixed brain tissue sections [38], this difference needs to be investigated further in in vitro study.

### 2.4. Summary of the Methodology

The use of human brain tissue has many challenges in terms of variability in agonal physiological conditions, postmortem delay, tissue availability, and differences between human subjects, but these cells have roles in the development of relevant models for aging-associated human diseases. The message for researchers is that human microglia in culture are feasible models for human neuroinflammation if consistent procedures for isolation and culture are used. These isolated cells are phenotypically heterogeneous, so experimental comparisons using cells from different preparation procedures and brain regions may produce discordant results. However, as will be discussed in the following sections, using cells prepared from enough cases using the same procedure and brain regions, one can study the effect of age, disease, and treatment on microglial phenotypes and functions and even the effects of disease can be maintained in the cultured cells.

## 3. Properties of Human Microglia in Culture

### Human Microglia in Culture: How Much Do They Change Their Properties?

**Figure 2:**
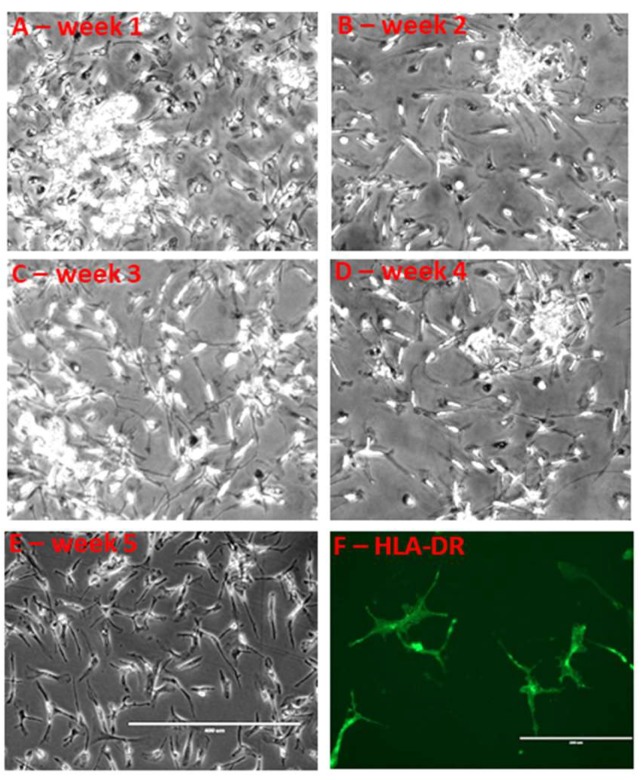
Long-term culture of human brain microglia. (**A**–**E**). Morphology of human brain microglia from ND (non-demented control case) over a 5-week period after replating. The total time in culture was a further 10 days since isolation from tissue. (**A**). One week after replating (total time in culture 17 days). (**E**). Five weeks after replating (total time in culture 45 days). (**F**) Microglia cultured for 5 weeks after replating and stained for microglial activation marker HLA-DR. Panels (**A**–**E**) have a same magnification; the scale bar on panel E represents 100 μm. Panel F has a higher magnification; the scale bar represents 50 μm.

There have been commentaries about the validity of culture models of human microglia as representations of in vivo microglia due to lack of purity of isolated cell types or phenotypic changes resulting from culture conditions. One concern that was considered when producing microglia from human brain tissue was the possibility that cultures would contain significant numbers of blood-derived macrophages. Blood macrophages, however, are significantly different in morphology, remaining as round cells, while cultured human microglia develop prominent processes similar to the morphologies seen in intact human brain tissues [7,8,11,34]. In addition, microglia can maintain viability in culture for a number of weeks, while macrophages cannot. Figure 2 shows the morphology of human microglia over a 5-week period in culture after isolation. The morphological features and stability of microglia are dependent on growth or maintenance conditions. For the experiments, we used a reproducible paradigm of culturing the microglia for 10–12 days in media containing 10% fetal bovine serum. During this period, they develop from small round cells into the typical microglia morphology (Figure 2A–E). We routinely characterized the microglia phenotype and the purity of each isolate by immunocytochemistry at the time of replating cells for the experiments. An example of immunofluorescence-labeled HLA-DR expression by 5-week-old cultured microglia is shown in Figure 2F.

The validity of experimental results employing activation or inhibitory agents is dependent on not changing the phenotypic properties of microglia during the culture and replating period. As microglia respond to inflammatory stimuli, when carrying out experiments, it is important to have high-quality endotoxin-free reagents or these reagents will produce artefactually-activated cells. For the experiments, the cells were removed from the primary culture vessel with 0.25% trypsin or the recombinant enzyme TrypLE (Thermo Fisher Scientific, Waltham, USA) and replated into the experimental numbers. One day post-plating, the growth media was replaced with serum-free media prior to stimulation with Aβ or other agents. In the absence of serum, microglia develop a more ramified morphology similar to brain resting microglia. This experimental paradigm, or slight modifications of it, have been used by the authors from 1995 to more recent publications [7,31], with reproducible results.

Phenotypic changes due to isolation and culture conditions need to be considered. Recent transcriptomic analysis of human microglia isolated from surgical tissues showed a culture interval-dependent gene induction and gene downregulation [39]. The human microglia were defined by live/DAPI − CD11b + CD45LowCD64 + CX3CR1High single cells. Microglia in culture for 6, 24, and 148 h were supplemented with 20 ng/mL IL-34. Under these culture conditions, partial recovery of the acutely up- and down-regulated gene changes were observed 7 days in vitro. Among these induced genes were those related to inflammation and stress responses. The largest change observed with culture was the increased mRNA expression of CD14, a lipopolysaccharide (LPS) receptor, which for a single case increased approximately 20-fold after 4 days in culture, while Toll-like receptor (TLR)-4 and interferon gamma (IFNγ) receptor 1 mRNA were not altered [36]. Subsequent analysis of a larger number of samples confirmed average CD14 mRNA upregulation by approximately two-fold, but with the downregulation of purinergic receptor (P2RY12), fractalkine receptor (CX3CR1) and complement receptor CD11b [26]. The expression of P2RY12 has been used as a marker for resting microglia but not macrophages [40]. This study showed the downregulation of a range of immune-related genes, including tumor necrosis factor (TNF)-α, HLA-DRA, immunoglobulin receptor CD16a, glutamate aspartate transporter (GLAST), transforming growth factor β, and interleukin (IL) 10, but not IL 1α or CD45. Although microglia undergo some phenotypic changes in culture, it is clear that they are not activated in a traditional proinflammatory sense. We have shown that even after 14 days in culture, microglia do not produce high levels of inflammatory cytokines unless activated by stimuli [41]. Although Mizee and colleagues [37] have shown downregulation of P2RY12 mRNA with 4 days in culture (suggesting activation), we have observed its stable expression in 10–14-day cultured microglia compared to similarly cultured human macrophages (RNA sequencing data from [28]). Human blood-isolated macrophages have been used in our laboratory and others as the cell type suitable for comparison with human brain-derived microglia [11,28,32,36].

## 4. Experimental Findings on Alzheimer’s Disease Using Human Autopsy Microglia

### 4.1. In Vitro Responses of Human Microglia to Aβ

The major focus for developing new treatments for AD has been preventing the formation and accumulation of Aβ [42,43]. Aβ in aggregated oligomeric and fibril forms can have multiple biological properties, including neurotoxicity and the activation of microglia [9,44,45]. The theme of most AD-related in vitro microglial studies has been responses to Aβ peptide (either recombinant or synthetic), particularly aggregated (fibrils or oligomers) Aβ (1–42), the abundant form found in amyloid plaques in AD brains. It was hypothesized and confirmed in a number of experiments using in vitro cultured human microglia that Aβ-activated microglia can produce toxic pro-inflammatory cytokines, ROS and neurotoxic factors [9,17,46,47]. The identification of human microglia-derived neurotoxins induced following stimulation with plaque material isolated from postmortem human brains was demonstrated a number of years ago, but these findings still require replication [48,49]. A prominent early response of microglia to proinflammatory stimulation is the activation of the NADPH oxidase complex, which leads to the excessive production of ROS. This complex is already formed and activation leads to the assembly of proteins and not new gene expression. Aβ can activate the NADPH oxidase complex of microglia to produce ROS [50]. There is evidence of increased NADPH oxidase activation in AD brains by biochemical methods [51]. ROS can be potentially damaging, but cells also produce a number of oxidases to reduce the consequences of activation.

Two types of experimental models with Aβ were employed in our microglia studies. Firstly, the more common paradigm of adding Aβ peptides to the culture media of microglial cultures, and secondly, the creation of in vitro plaques by depositing aggregated Aβ on culture plate surfaces. The latter has been used in experimental studies to reflect how microglia might chronically interact with aggregated Aβ, as these experimental studies can be carried out over a number of days rather than short-term. We used droplets containing highly-aggregated recombinant Aβ (1–42) solution air-dried on poly-lysine coated surfaces, followed by plating microglia into these culture wells [52]. This model was used to show the involvement of the receptor for advanced glycation endproducts (RAGE) in mediating microglial responses to Aβ (1–42) and AD-related neuroinflammation. Blocking microglial-expressed RAGE with F(ab)’_2_ fragments of antibodies to RAGE was able to reduce microglial migration towards Aβ deposits and also reduce the Aβ induction of macrophage colony-stimulating factor (M-CSF), also known as colony-stimulating factor-1 (CSF-1), secretion by microglia [53]. Activated microglia expressed increased amounts of cell-surface RAGE following stimulation with RAGE ligands, including Aβ (1–42).

This model was also used to investigate the responses of human microglia to Aβ antibody-opsonized in vitro plaques [41]. Earlier work has shown that microglia were more able to phagocytose and remove Aβ if it had been opsonized with Aβ-specific antibodies, both in vivo in experimental AD mice model and in vitro in rodent and human microglia [54]. Using the in vitro amyloid plaque model, we showed that precoating them with antibody to Aβ (6E10) resulted in enhanced phagocytosis of Aβ by human microglia, as expected, but also resulted in enhanced inflammatory responses by the increased secretion of cytokines and chemokines [41]. As microglia express all three types of immunoglobulin Fcγ receptors (CD16, CD32 and CD64), engagement of these receptors by the Fc portions of Aβ antibodies could not only stimulate microglial phagocytosis but also activate different, possibly damaging, proinflammatory pathways. The use of peptides to produce an Aβ-antibody response or administration of antibodies to stimulate Aβ plaque removal has been extensively tested in clinical trials involving active and passive immunotherapy. The initial trials of active immunization with Aβ peptide, which showed promise in transgenic mice, were found to cause meningoencephalitis in human subjects, resulting in this approach being discontinued [55,56]. Of the peptide immunized human subjects that subsequently underwent neuropathological examination after autopsy, some showed that much of the plaque material had been effectively removed, presumably by activated microglia [57,58,59]. In recent years, multiple trials of passive immunotherapy with monoclonal antibodies against various Aβ epitopes have been conducted, but the effectiveness of this approach remains unresolved, suggesting that the way brain microglia respond to antibody-opsonized Aβ plaques in early subjects is not adequately understood.

### 4.2. Human Microglial Responses to Aβ (1–42) Peptide

In an earlier study, we showed that Aβ stimulated the expression of urokinase plasminogen activator receptor (uPAR) by cultured microglia [60]. This response was sensitive to free radicals being inhibited by antioxidants. The upregulation of uPAR has been reported as a significant activation marker for macrophages and microglia [61,62]. These in vitro results correlated with increased uPAR expression in AD brains in hippocampus, inferior temporal gyrus and superior frontal gyrus [60]. This study was extended to include gene expression profiling of larger numbers of inflammatory genes of microglial responses to Aβ peptide [9]. This study provides a large amount of data on all cellular responses affected by this treatment. One of our earlier studies that used membrane arrays for a limited number of genes showed that multiple inflammatory genes were upregulated in human microglia after 24 h of treatment with Aβ (1–42) [63]. The experiments were originally designed to detect the chronic effects of Aβ stimulation, but this time period may have been too short for this and was still detecting acute responses. In a follow-up study, expression profiling was carried out with gene arrays that covered the complete human transcriptome of approximately 30,000 targets. Multiple inflammatory genes were activated at 24 h, the most abundant being IL1B [9]. On average, across the 5 samples analyzed, the top-10 induced genes were IL1B (80.3 fold), IL8 (62-fold), CCL20 (51.1-fold), GOS2 (47.6-fold), CXCL1 (46.0-fold), IDO (44.4-fold), MMP1 (39.6-fold), MMP3 (31.4-fold), IL1A (27-fold) and CCR7 (24.4-fold). We also noticed that a number of genes were downregulated after stimulation. Some of these genes were involved in normal microglial function but some also in immune function. The top-10 downregulated genes were LIPA (20.6-fold), SEPP1 (13.4-fold), HLA-DMR (13.3-fold), IFIT2 (11.6-fold), TREM2 (8.2-fold), SDF1 (7.4-fold), MNDA (7.2-fold), LMO2 (6.6-fold), A2M (6.5-fold), and SDR1 (6.3-fold). The downregulated genes showed that a certain amount of cellular reorganization occurred in response to proinflammatory activation. Although the changes were of less magnitude, it was noticeable that a number of genes related to microglial phagocytosis were either downregulated or unchanged following Aβ (1–42) stimulation.

### 4.3. Human Microglial Responses: Differences Between AD and ND Microglia

A study using microglia from a series of cases diagnosed as non-demented (ND) or AD measured the constitutive expression and Aβ-induced expression of complement and cytokine genes. This study showed that complement C1q and CSF-1 were secreted constitutively in higher amounts in microglia derived from AD brains [64]. This finding showed that some of the disease features of activated microglia from AD brains were maintained even after 2 weeks in culture. A difference in calcium-mediated signaling responses was also observed between AD and ND brain-derived microglia [65]. AD microglia had higher basal calcium levels, diminished responses to ATP and platelet activation factor (PAF), which is indicative of a depletion of calcium from the endoplasmic reticulum, and diminished amplitude store operated calcium channel responses to ATP and PAF. The global gene expression profiling of AD and ND microglia identified ADM, BMP6, CXCL1, ILRA and VDR to be induced at significantly higher levels by Aβ peptide in AD compared to ND microglia [9]. The disease effects on the phenotypes of isolated cultured cells were also reported in a recent transcriptome study [39]. When comparing microglia transcriptome and whole-brain cortical gene profiles associated with AD, Parkinson’s disease, multiple sclerosis, and schizophrenia, the researchers found largely non-overlapping profiles that exhibited preferential expression corresponding to the disease [39].

### 4.4. Using Human Microglia to Study Inflammation Control Pathways Relevant to AD

A number of different cellular inflammation regulatory systems exist to limit the consequences of proinflammatory activation by microglia/macrophages. Some of these have been studied in relation to AD pathogenesis using human microglia. Of particular interest was the CD200 receptor (CD200R), expressed by macrophages/microglia and its ligand CD200 [32,66]. CD200 is abundant on neurons and myelinated structures, and the expression of CD200R in the brain is restricted to microglia. The activation of CD200R by CD200 results in the downregulation of activation by microglia. This system appears to be deficient in AD and MS brains, but not in brains primarily affected by α-synuclein-mediated Lewy body diseases [32,66,67]. A feature observed in AD and MS brains is that CD200R expression by microglia is very low, possibly with insufficient expressed to be functional, however, its expression was increased in response to the anti-inflammatory cytokines IL-4 and IL-13, making it a marker of alternative (M2a) activation [32,66,68]. The low levels of CD200R mRNA expression in microglia were confirmed in microglia analyzed directly after isolation from human brains [35,69]. Another class of regulatory proteins are suppressors of cytokine signaling (SOCS) proteins. These are a class of seven related proteins that function to deactivate inflammatory cytokine signaling by promoting the degradation of cytokine or growth factor-activated JAK/STAT complexes. The most widely studied of these have been SOCS-1, -2, and -3, with SOCS-3 induction being associated with macrophage activation. Using postmortem microglia, we showed that SOCS-1, -2, and -3 mRNA were significantly upregulated in Aβ-stimulated microglia as well as in AD brains. By contrast, SOCS-6 was downregulated with immune stimulation and in AD brains [70]. These findings provide evidence of complex control mechanisms for inflammation present in brain and microglia that are working to prevent excess of damaging inflammation.

### 4.5. Human Microglia and SNPs Associated with Altered Risk of AD

A number of the new genetic risk factors for AD identified from genome-wide association studies (GWAS) have been single nucleotide polymorphisms (SNP) associated with microglial or inflammation-related genes [71]. These new discoveries have renewed interest in inflammatory mechanisms for AD [72]. The two most-prominent AD risk-associated SNPs are triggering receptor expressed on myeloid cells (TREM)-2 and the sialic acid-binding protein CD33. TREM-2 SNP (rs75932628), which results in a genetic mutation in the protein (R47H), is rare but results in a higher risk of AD, possibly due to the loss of function [71,73]. In our study of TREM-2 in brain tissue, we showed increased expression of TREM-2 in AD brains with expression restricted to microglia, particularly those around Aβ plaques [74,75]. However, stem cell-derived macrophages and microglia with the R47H mutation were recently shown to have no loss of phagocytic activity [76]. TREM-2 has been shown to be a receptor for lipids, apolipoprotein E, Aβ and bacteria [77,78,79].

The CD33 SNP (rs3865444) is located in the upstream non-coding region and controls the transcription of the gene. The common A/A phenotype causes a reduced risk of AD [80]. Similar to TREM-2, CD33 also has expression restricted to macrophages or microglia in AD brains [74,81]. The role of CD33 function in AD and AD-related neuroinflammation has not been established. CD33 is an anti-inflammatory immune receptor regulator; the interaction of CD33 by sialic acid-conjugated lipids or proteins activates inhibitory signaling pathways including those related to phagocytosis. Higher levels of CD33 expression by microglia are believed to reduce Aβ phagocytosis, while lower levels of expression in A/A SNP phenotype result in more phagocytosis [82]. There is no evidence that CD33 is an Aβ phagocytic receptor, but its activation could alter the activity of other phagocytic receptors. We showed that CD33 expression by human microglia was significantly downregulated by most stimulating agents, including Aβ, LPS and inflammatory cytokines, but not TNF-α [81]. CD33 functions to reduce inflammatory signaling with reduced CD33 expression in the monocytes of diabetic patients, correlating with enhanced inflammation [83]. As controlling inflammation in AD brain is considered a key therapeutic target, the loss of CD33 inhibitory signaling may actually have adverse consequences, even if this results in increased Aβ phagocytosis. This will need to be determined in further studies.

Progranulin, which is considered as a neurotrophic factor with significant anti-inflammatory properties, has been observed to be increased in activated microglia in intact AD brain tissue [84,85]. The SNP rs5848 T/T variant that affects the levels of the expression of progranulin mRNA has been associated with an altered risk of AD [86,87]. Progranulin is expressed by microglia and neurons in the brain. One study measured the expression and secretion by human fetal brain-derived microglia. This study showed in vitro that progranulin mRNA and protein expression and secretion by microglia was downregulated by proinflammatory agents LPS and IL-1β/IFN-γ but upregulated by IL-4 and IL-13. These results suggest that increased progranulin expression in vivo was associated with alternative (M2a) microglia/macrophage activation [88].

### 4.6. Aging Human Microglia

One advantage of using postmortem human microglia derived from elderly brains is that they may carry aging-related transcriptomic patterns appropriate to their age. Recently, in a study comparing gene expression in mid-age (mean of age was 53 ± 5.2) and old-age (94.07 ± 0.95) subjects, Olah et al. identified upregulation of 1060 genes and downregulation of 1174 genes in microglia isolated from old-age subjects [89]. Intriguingly, amyloid formation-related gene sets were enriched in upregulated genes, whereas homeostasis-related genes of the TGFβ signaling KEGG pathway were enriched in the downregulated genes. These findings suggest that aging alone could compromise the maintenance of homeostasis by microglia and increase the accumulation of amyloid. Age-induced changes in the microglia of genes involved in actin assembly, axonal guidance, cell adhesion, chemotaxis, and sensome have been observed [90]. These transcriptomics technology-based findings reveal the scope of aging effects on human microglia functions, providing insight into how the physiological age of microglia might be a critical factor to be considered when used as experimental models. 

## 5. Enhancers of Proliferation, Cell Transformation and Microglial-Derived Induced Pluripotent Stem Cells (iPSCs)

In the preceding sections of this article, we outlined the methodology and some of the valuable findings from human brain-derived microglia, as well as the feasibility of isolating human autopsy brain microglia for experimental studies. However, primary microglia from autopsies are often limited by the availability of postmortem brain materials to many researchers. Three approaches to providing increased numbers of microglia, or reliable and sustainable sources have been the use of growth factors to the induced cellular proliferation of microglia, the transformation of primary cells into immortal cell lines, and the production of adult microglia from induced pluripotent stem cells (iPSCs). We will discuss these approaches in the following section.

### 5.1. Growth Factors for Inducing Cell Division in Human Microglia

Human microglia—especially from postmortem tissue—for use in experimental studies have limitations in the numbers of cells that can be obtained as they do not proliferate. This is particularly an issue when compared with the numbers of microglia that can be grown from neonatal rodents. A number of studies have examined how to increase cell numbers with the use of macrophage growth factors. This approach has been successful to some extent, but before growth factors are used for experimental purposes, whether or not the use of growth factors will change the activation phenotypes of cultured cells must first be considered. Based on our experience with postmortem brain-derived microglia, we found that isolated cells showed very little cell division when cultured in regular media containing only fetal bovine serum. The addition of CSF-1 alone to media had minimal effects, but the recently discovered cytokine/growth factor IL-34, which shares the CSF-1R receptor with CSF-1, caused a robust induction of microglial cell division [28]. As part of this, we investigated whether IL-34 induced a different gene expression profile in human microglia than in CSF-1. Although we did not identify differences in gene expression signatures between the CSF-1 and IL-34 treatments, the effect of IL-34 alone on microglial cell division was consistent. Therefore, the mechanism for this specific response remains undetermined. An earlier study by other researchers using human fetal and adult microglia showed that CSF-1 could induce some cell division, while granulocyte macrophage-colony stimulating factor (GM-CSF or CSF-2) was significantly more effective [91]. These authors showed a synergistic effect on cell division if both cytokines were added to cell cultures. Another report on this topic describes the continued passage of human postmortem brain microglia multiple times (up to 17 times) while apparently maintaining their phenotype. These cells maintained the expression of CD68, ROS production, phagocytic responses to Aβ and other ligands and expression of ligand DiI-Ac-LDL [33]. The authors prepared microglia from the brain using only gray matter in a similar manner to our protocol but did not use the density-gradient centrifugation step. The growth media reported to be used to obtain this level of cell division contained GM-CSF and a proprietary “microglial growth supplement” from a commercial source (ScienCell Laboratory, San Diego, CA, USA). The nature of this supplement is not known, but it likely contains a mixture of CSF-1 and IL-34 to have such an effect. 

### 5.2. Transformed Microglial Cell Lines

The transformation of human microglia into immortal, proliferating cells has been achieved by the transfection or transduction of oncogenes. The use of SV40 T antigen for the transformation of microglia has produced cell lines, but these seem to lose their differentiated microglial properties when cultured. An example of such cells is the CHME-5 cell line [92]. A human microglial cell line (designated HMO6), transformed from fetal microglia with a retrovirus vector encoding c-myc oncogene, was produced and characterized [13,93]. These cells appeared to maintain a stable phenotype over a number of years and respond to inflammatory stimuli with expression of a range of cytokines and cell type-specific antigens such as CD11b (Mac-1), CD68, CD86 (B7–2), HLA-ABC, HLA-DR, and RCA-1 lectin, and actively phagocytose latex beads. This cell line has been used mostly by the originating laboratory in studies on microglial functions, including Aβ uptake and degradation [94], demonstrating similar properties to primary human microglia. These results have not been replicated, as there has been limited distribution of these cells to other academic researchers. Recently, Garcia-Mesa and colleagues (2017) achieved the transformation of human biopsy brain-derived microglia with a SV40 T antigen vector, with these cell lines maintaining many of the properties of adult microglia. Microglia transformed by SV40 T antigen alone replicated rapidly, while cells transformed with SV40 T antigen and human Telomerase reverse transcriptase (hTERT) vector maintained a slower-dividing and less-activated phenotype [95]. It is not clear from the publication how many cell divisions/passages were performed with these transformed microglia, so the stability of the phenotype still needs to be established in further studies. 

### 5.3. Human Microglia-Like Cells Produced from iPSCs

Recently, genetic reprogramming of adult somatic cells into iPSCs that can be differentiated into all other cell types has progressed to allow the reproducible production of “adult human microglia”. Previous studies have described different methods of producing cells with the properties of human microglia with different combinations of differentiation/maturation factors being used between these different methods to achieve the same outcome [96,97,98,99]. Detailed descriptions of these methods require reference to the original papers, but one method appears to be the most suitable for application by other researchers due to its relatively straightforward protocol [96,99]. In the original report of this method, iPSCs were transformed into hematopoietic progenitor cells over an 11-day period and then were cell-sorted by FACS or magnetic selection to select for CD43+ cells. These cells were then treated with microglial differentiation factors, including CSF-1, IL-34, TGFβ and insulin, for 25 days, and then with microglial maturation factors CD200 and CXCL1 (fractalkine) for 3 days to produce the adult human microglia [96]. These cells were shown to have similar gene expression profiles and properties to primary human adult microglia. A modification of this procedure showed that the cell-sorting stage could be omitted by removing the non-attached cells and treating them using the microglial differentiation protocol and discarding the attached cells [99]. This procedure has been adapted to prepare a commercial source of iPSC-derived microglia. It is now possible to purchase hematopoietic progenitor cells with which to create differentiated microglia, or alternatively to purchase already-differentiated microglia (Fuji Film/Cellular Dynamics International, Madison, WI, USA) (https://fujifilmcdi.com/assets/FCDI_iCellMicroglia_UG.pdf or https://fujifilmcdi.com/assets/CDI_iCellHematopoieticProgenitorCellsPrototype_UG.pdf). The commercially-sourced microglia still require maintenance media containing CSF-1, IL-34, TGFβ, CD200 and CXCL3 to complete the differentiation. The inclusion of maturation factors CD200 and CXCL3 came from earlier observations that to be fully mature, microglia require interaction with neurons or neuronal-derived proteins to achieve their characteristic phenotype. Both factors are expressed predominantly by neurons in brains. However, another iPSCs-derived microglia protocol does not require CD200 and CXCL3. In this protocol, cells were first selected from myeloid progenitor cells by selecting for CD14 and CX3CR1 (fractalkine receptor) expression prior to their differentiation into microglia using GM-CSF and IL-34 [100].

The potential applications of iPSCs-derived human microglia, especially if they can be produced in large numbers, are broad [101]. The microglia produced by one of the methods mentioned above [96,99] reportedly have gene expression profiles that are highly similar to human fetal and adult brain-derived primary microglia, showed Aβ phagocytosis and chemotactic responses, could be used in in vitro coculture studies, and remained functional when transplanted into AD model mice. These cells were shown to have a type-I interferon response to the anti-viral drug Gangciclovir similar to the one observed in murine microglia [102].

One particular advantage of iPSC-microglial technology over primary human microglia isolates is the ability to more readily produce cells from subjects with known genetic mutations in inflammation-associated genes, although it is also possible to obtain primary microglial cultures from the autopsies of subjects prescreened for genetic SNPs or mutations.

Recent studies have reported on the properties of microglia derived from iPSCs with TREM-2 mutations. Human microglia were produced from iPSCs obtained from subjects with TREM-2 disease-associated missense mutations in T66M and W50C by differentiation with BMP4, SCF and VEGF for 4 days, followed by CSF-1 and IL-3, with microglial differentiation induced by IL-34 and GM-CSF (34 days total) [76]. Microglia produced by this iPSCs differentiation method were compared with those prepared by another protocol [96] and were shown to have very similar RNA expression profiles. In microglia derived from TREM-2 mutation cases, TREM-2, accumulated in an immature form, did not undergo normal proteolysis and was not properly trafficked to the plasma membrane, making it non-functional. These microglia, however, did respond to LPS and were functionally phagocytic like the microglia from non-TREM-2 mutation cases. A similar finding was obtained in another study using iPSCs-derived microglia from control, T66M and W50C TREM-2 mutations [103]. Microglia without mutations shed TREM-2, while those from mutation cases produced significantly reduced amounts of soluble TREM-2. The total expression levels of TREM-2 mRNA or protein in microglia derived from mutated cases were significantly lower than microglia without mutations [103]. In this study, the TREM-2 mutation-derived microglia responded to LPS, comparably to non-mutation control microglia, but did show deficits in phagocytic activity.

It is well established that possession of the apolipoprotein E4 (APOE4) allele is the most significant risk factor for developing AD, but the nature of the cellular and pathological mechanisms affected by this protein compared to APOE3 producers are still unclear [104,105]. The APOE4 allele was recently shown to potentially act synergistically with the R47H TREM-2 variant in the pathogenesis of AD [106]. One recent study produced neurons, astrocytes and microglia from iPSCs derived from APOE4 and APOE3 homozygous subjects and demonstrated the genotype-dependent properties of these cells [107]. The gene expression of all cell types with homozygous APOE4 was more similar to an AD phenotype than that of cells carrying only the APOE3 genotype. APOE4 neurons had increased numbers of synapses, early endosomes, and increased secretion of Aβ, while APOE4 astrocytes had a reduced secretion of apolipoprotein E protein and a reduced uptake of Aβ. The phenotype of homozygous APOE4 iPSCs-derived microglia was also noticeably different from homozygous APOE3 cells. It had a more activated gene expression profile, a reduced Aβ uptake and less complex process formation. This study utilized these cells in a 3D cerebral organoid model that studied microglia interacting with neurons and astrocytes. APOE4 organoids of neurons and microglia had higher levels of Aβ and an increased intensity of phosphorylated tau. This study also shows that converting APOE4 to APOE3 using CRISPR/Cas9 gene-editing technologies resulted in significant attenuation of AD phenotyping in individual cells and organoids. This experimental approach with human cells offers great promise in identifying new therapeutic approaches.

TREM2 phagocytosis function has also been investigated in transdifferentiated microglia-like cells (tMGs) obtained from monocytes differentiated from human iPSCs) that expressed TREM2^+/R47H^, TREM2^+/−^, and TREM2^−/−^ hPSCs using CRISPR/Cas9 [108]. Phagocytosis was examined on amyloid plaques present in cryo-sections cut from 6-month-old APP/PS1^+/−^ mouse brains. The phagocytic functions were lower in TREM2^+/−^ and TREM2^−/−^ tMGs than in wild-type tMGs or TREM2^+/R47H^ tMGs. 

As described above, the effects of TREM2 variants, including R47H, T66M, and W50C, on phagocytic functions were studied in the mutant carriers or genetically engineered iPSCs-derived microglia. Although aging and epigenetic signatures are erased during the production of iPSCs, the success of generating iPSCs-derived microglia does provide tremendous opportunities for using human microglia in experiment models and overcomes the limitation related to the accessibility of human postmortem microglia.

## 6. Conclusions

Experimental systems using human microglia cultures have advanced the understanding of microglia biology and their roles in health and disease. This review focused on the utility and the findings of human microglia studies, including primary microglia and microglia differentiated from iPSCs. The potential for future research in this field to advance the understanding of microglia, neuroinflammation, and identifying therapeutic targets for AD is significant. While most of the research will still include models of inflammation bases on rodents, there is a place for human in vitro microglia in any discovery scheme. Although iPSCs-derived human microglia may be able to substitute for primary human microglia, there is still relevance for primary human microglia derived from postmortem human brain tissue due to their closer resemblance to human microglia in vivo for target validation in investigations of therapeutic approaches for AD. These cells reflect the phenotypes resulting from exposure to many years of environmental and genetic factors from real life, which has been increasingly acknowledged as important for understanding the failure of disease-modifying clinical trials. Immortalized cell lines offer reproducible models and they cannot recapitulate these genetic and the epigenetic heterogeneity of aging human disease. Postmortem microglia also offer the opportunity to study microglia heterogeneity based on the brain region of origin, about which we currently know very little [109]. As microglial-driven neuroinflammatory responses play major roles in the pathogenesis of AD and other neurodegenerative diseases, multiple experimental approaches will continue to offer synergism in furthering our understanding and guiding the design of therapeutic innovations.
